# Supplementary material for: The interactome of intact mitochondria by cross-linking mass spectrometry provides evidence for coexisting respiratory supercomplexes
Source: Mol Cell Proteomics. 2017 Dec 8;17(2):216–32. doi: 10.1074/mcp.RA117.000470 (PMC5795388; doi:10.1074/mcp.RA117.000470)
Supplement: Supplemental Data [file supp_RA117.000470_133922_0_supp_23978_fzffwf.zip › spectra_annotation/mito_spectra_annotation/359-1-7-1-13-1.pdf]

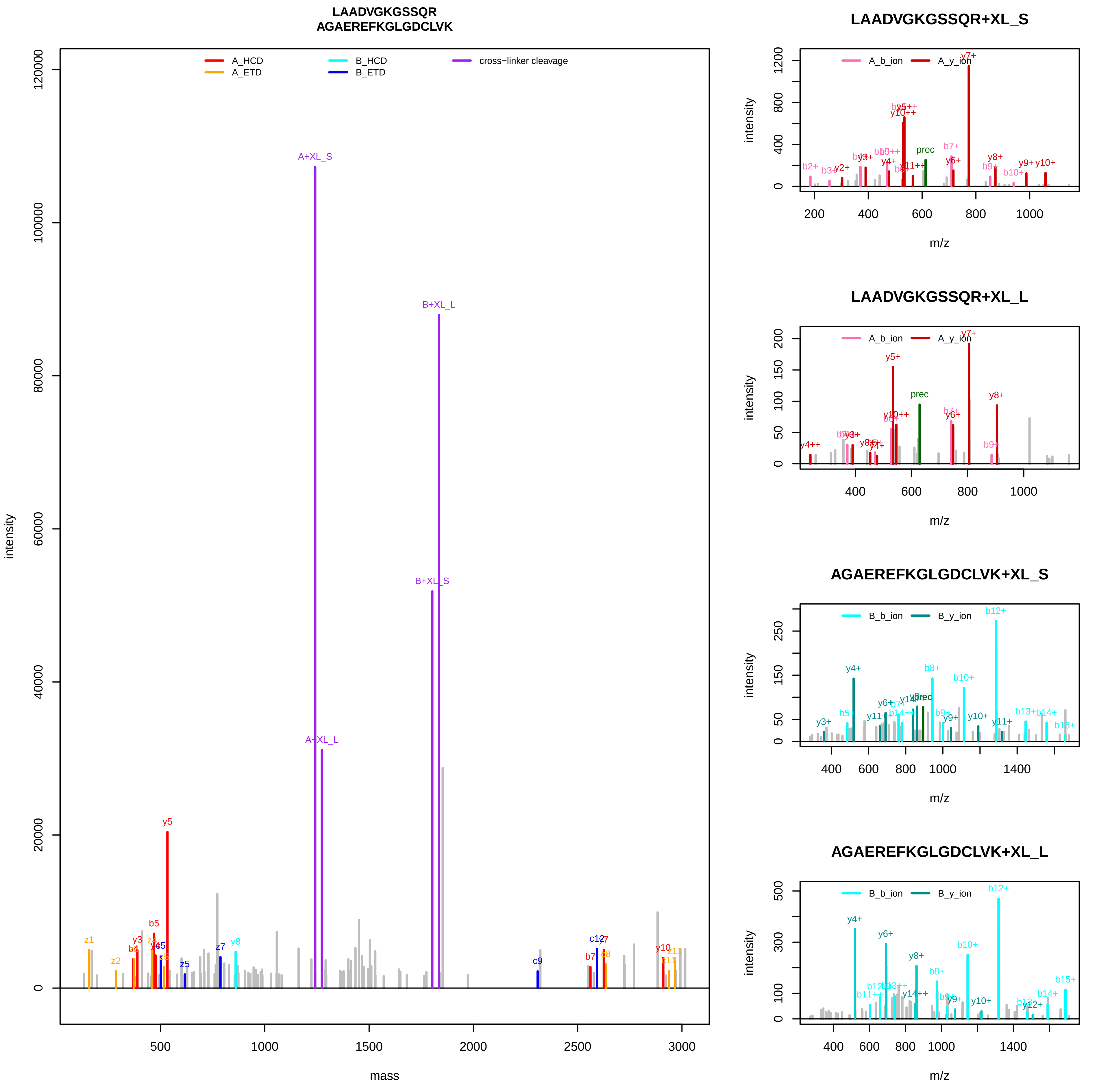

LAADVGKGSSQR
AGAEREFKGLGDCLVK
A_HCD
A_ETD
B_HCD
B_ETD
cross−linker cleavage
A+XL_S
B+XL_L
B+XL_S
A+XL_L
y5
b5
z1
z2
y3
b4
c5
z5
z7
y8
c9
c12
y7
b7
z8
y10
z11
c11
intensity
mass
0
20000
40000
60000
80000
100000
120000
500
1000
1500
2000
2500
3000
LAADVGKGSSQR+XL_S
A_b_ion
A_y_ion
y7+
y10++
y5+
prec
b7+
y8+
y9+
y10+
b10+
b2+
b3+
y2+
y3+
y4+
y6+
b9+
m/z
LAADVGKGSSQR+XL_L
y5+
prec
y8+
y10++
y6+
y4++
y3+
b9+
AGAEREFKGLGDCLVK+XL_S
B_b_ion
B_y_ion
b12+
y4+
b8+
b10+
y6+
b7+
b5+
y3+
y9+
y10+
y11+
b13+
b14+
b15+
AGAEREFKGLGDCLVK+XL_L
b12+
y4+
y6+
y8+
b8+
b10+
b15+
b14+
y10+
y9+
y12+
